# Distinctive tumorigenic significance and innovative oncology targets of SUMOylation

**DOI:** 10.7150/thno.97162

**Published:** 2024-05-19

**Authors:** Heng Zhou, Na Deng, Yanshu Li, Xiaoyun Hu, Xue Yu, Shiheng Jia, Chen Zheng, Shan Gao, Huizhe Wu, Kai Li

**Affiliations:** 1 Department of Surgical Oncology and General Surgery, The First Hospital of China Medical University, Shenyang, Liaoning 110001, China; Key Laboratory of Molecular Pathology and Epidemiology of Gastric Cancer in the Universities of Liaoning Province, Shenyang, Liaoning 110001, China.; 2 Department of Anesthesiology, The First Hospital of China Medical University, Shenyang, Liaoning 110001, China.; 3 Department of Hematology, The Fourth Affiliated Hospital of China Medical University, Shenyang, Liaoning 110001, China.; 4 Department of Cell Biology, Key Laboratory of Cell Biology, National Health Commission of the PRC and Key Laboratory of Medical Cell Biology, Ministry of Education of the PRC, China Medical University, Shenyang, Liaoning 110122, China.; 5 Scientific Experimental Center, School of Pharmacy, China Medical University, Shenyang, Liaoning 110122, China.; 6 Department of Gynecology and Obstetrics, Shengjing Hospital of China Medical University, Shenyang, Liaoning 110001, China.; 7 Department of Pharmacology, School of Pharmacy, China Medical University, Shenyang, Liaoning 110122, China.; 8 Liaoning Key Laboratory of Molecular Targeted Anti-Tumor Drug Development and Evaluation; Liaoning Cancer Immune Peptide Drug Engineering Technology Research Center; Key Laboratory of Precision Diagnosis and Treatment of Gastrointestinal Tumors, Ministry of Education; China Medical University, Shenyang, Liaoning 110122, China.; 9 Shenyang Kangwei Medical Laboratory Analysis Co. LTD, Liaoning Province, China.

**Keywords:** Cancer, SUMOylation, Post-translational modification, Cancer hallmarks, Cancer therapy

## Abstract

Protein SUMOylation, a post-translational modification, intricately regulates diverse biological processes including gene expression, cell cycle progression, signaling pathway transduction, DNA damage response, and RNA metabolism. This modification contributes to the acquisition of tumorigenicity and the maintenance of cancer hallmarks. In malignancies, protein SUMOylation is triggered by various cellular stresses, promoting tumor initiation and progression. This augmentation is orchestrated through its specific regulatory mechanisms and characteristic biological functions. This review focuses on elucidating the fundamental regulatory mechanisms and pathological functions of the SUMO pathway in tumor pathogenesis and malignant evolution, with particular emphasis on the tumorigenic potential of SUMOylation. Furthermore, we underscore the potential therapeutic benefits of targeting the SUMO pathway, paving the way for innovative anti-tumor strategies by perturbing this dynamic and reversible modifying process.

## Introduction

SUMOylation, identified in the 1990s, is a dynamic and reversible protein post-translational modification 1. In a nutshell, this process is a multi-step enzymatic cascade catalyzing the covalent attachment of small ubiquitin-like modifier (SUMO) proteins to specific lysine (K) residues of substrate proteins 2. Typically, SUMOylation and ubiquitination compete for substrate proteins; however, unlike ubiquitination, which often targets proteins for degradation, SUMOylation primarily maintains protein stability. Dysregulation of SUMOylation has been validated across more than 10 types of tumors, including colorectal cancer (CRC) 3, lung cancer 4, and hepatic cancer 5. In most cases, SUMOylation exerts an oncogenic effect in cancers by modulating critical biological processes such as transcriptional regulation, protein-protein interaction, protein translocation, phase separation, and protein stability. Consequently, SUMOylation influences significant biological processes, involving gene expression, cell cycle progression, signaling pathway transduction, DNA damage response, and RNA metabolism, thus contributing to tumorigenicity and sustaining cancer hallmarks such as tumor invasion, metastasis, programmed cell death escape, metabolic reprogramming, tumor immune evasion, and epigenetic reprogramming. Additionally, diverse cellular stressors, such as hypoxia, viral infection, gut microbiota alterations, and lactic acid levels, contribute to the dysregulation of SUMOylation in tumor initiation and development. Furthermore, SUMOylation has been implicated in the development of multidrug resistance during tumor therapy 6. Thus, targeting the enzymes involved in SUMOylation, through the development of pharmaceutical inhibitors comprising natural and synthetic compounds, emerges as a crucial avenue for anti-tumor strategies. This review provides a comprehensive overview of the process and biological significance of SUMOylation in cancer, elucidates the cancer hallmarks maintained by SUMOylation, explores the cellular stressors triggering SUMOylation, and discusses the potential of targeting SUMOylation for anti-tumor therapy. By shedding light on the oncogenic role of SUMOylation, this review contributes to the exploration of new therapeutic strategies in cancer treatment.

## SUMOylation cascade and biological significance in tumors

### Overview of the SUMOylation process

SUMOylation involves multiple proteins, including SUMOs, SUMO E1 activating enzymes, SUMO E2 conjugating enzyme, SUMO E3 ligases, and SUMO-specific proteases. Among mammals, the SUMO family comprises five members: SUMO1, SUMO2, SUMO3, SUMO4, and SUMO5. Among these, SUMO2 exhibits the highest abundance, compensating for the reduced levels of SUMO1 and SUMO3 7, 8. Notably, SUMO2 and SUMO3 share a striking similarity in amino acid sequences, exceeding 95%, and are collectively referred to as SUMO2/3. Currently, the functionality of SUMO4 in substrate protein conjugation remains debated. While one study suggests that the unique proline-90 residue of SUMO4 may hinder its maturation, potentially impeding substrate conjugation 9, subsequent research has failed to provide conclusive evidence for the functional significance of the proline-90 residue of SUMO4 10. Recently, SUMO5 has emerged as a novel poly-SUMO isoform implicated in SUMOylation, enhancing the conjugation capacity of SUMO2/3 in human cells 11. However, its classification as a human SUMO pseudogene or a functional isoform necessitates further investigation.

**Figure 1:**
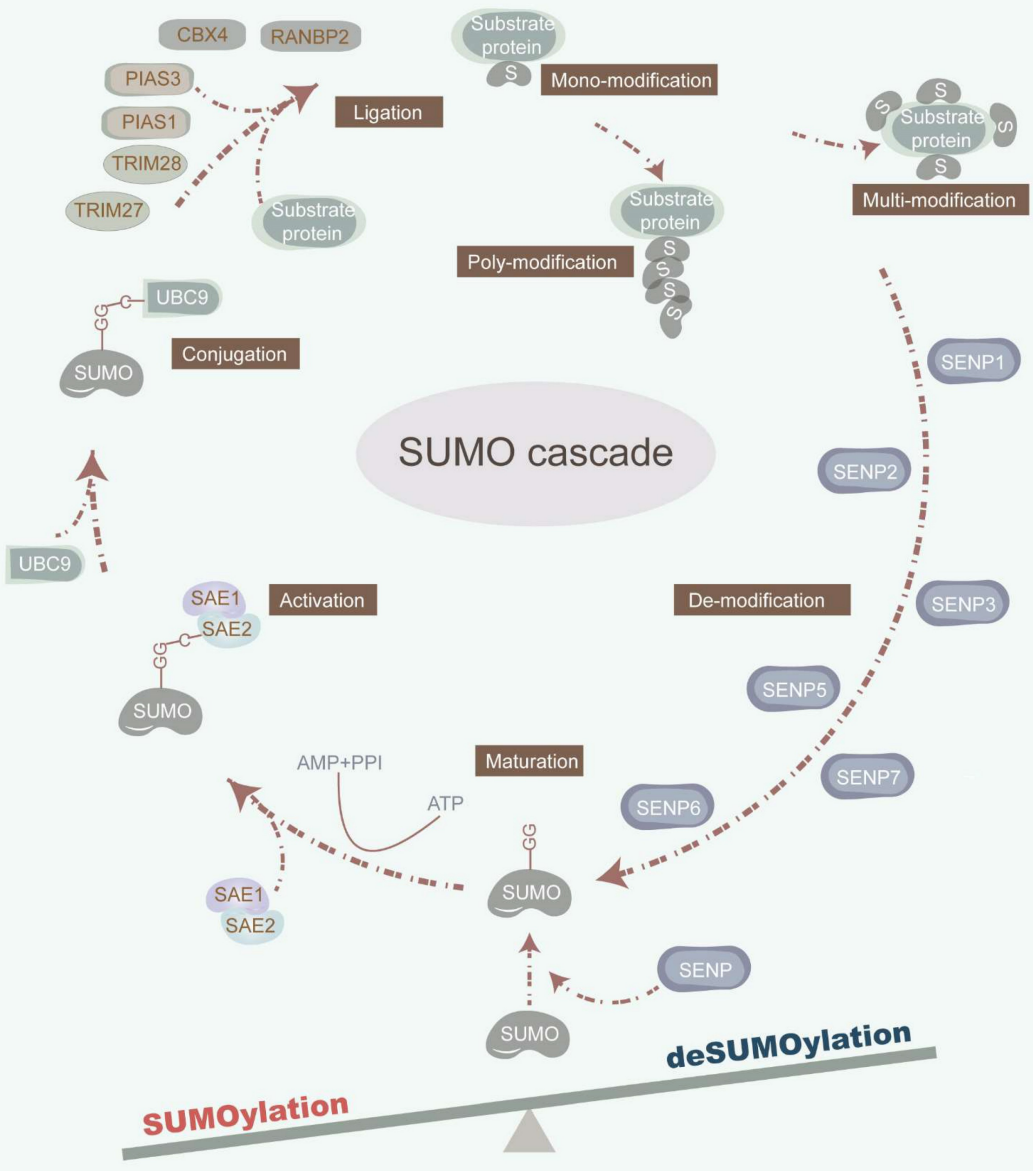
** The SUMOylation cascade.** SUMOylation is a multi-step enzymatic cascade catalyzing SUMOs covalently conjugating to substrate proteins. This dynamic and reversible SUMOylation process consists of multi-step actions, involving maturation, activation, conjugation, ligation, and deconjugation. SENPs cleave the amino acids in the C-terminal of inactive precursors of SUMO proteins to expose their diglycine (-GG) motif. SAE1/2 combines its cysteine site with the C-terminal of SUMO under the premise that ATP hydrolysis provides energy. Under the synergy of UBC9 and SUMO E3 ligase, the SUMOs are covalently conjugated to a lysine (K) in the SUMOylation consensus motif ψKx[E/D]. SENPs also orchestrate the deSUMOylation by removing SUMOs from substrate proteins.

The dynamic and reversible nature of SUMOylation involves a series of enzymatic steps, including maturation, activation, conjugation, ligation, and deconjugation 2 (Figure 1). Mechanistically, sentrin-specific proteases (SENPs) catalyze the proteolytic cleavage of the C-terminal amino acids of inactive SUMO precursor proteins, exposing their diglycine (-GG) motif and promoting maturation. The dimeric SUMO-activating enzyme E1 (SAE1/2) interacts with the C-terminal of SUMO, facilitated by ATP hydrolysis. Activated SUMOs are then transferred to a cysteine residue of ubiquitin carrier protein 9 (UBC9), a SUMO E2 conjugating enzyme. Finally, with the assistance of UBC9 and SUMO E3 ligase, SUMOs are covalently attached to a lysine (K) within the SUMOylation consensus motif ψKx[E/D] (where ψ represents a hydrophobic residue; x denotes any amino acid; K signifies lysine; E refers to glutamic acid; D refers to aspartic acid) or in the reversed motif [E/D]xK of target proteins. Of note, SUMO E3 ligases are primarily composed of the protein inhibitors of activated signal transducer and activator of transcription (PIAS) with SP-RING and SP-CTD domains, bind to UBC9 or SUMO, respectively 12. Furthermore, SUMO can non-covalently bind to proteins harboring SUMO-interacting motifs (SIM). The deconjugation of SUMOs is manipulated by SENPs, which also facilitate the maturation of SUMO precursors. Mammals possess six SENP family members, including SENP1-3 and SENP5-7, each exhibiting distinct activities toward individual SUMOs, which orchestrate the deSUMOylation by removing SUMOs from substrate proteins 13. For instance, SENP1 displays heightened activity on SUMO1, while SENP2 prefers SUMO2 14. Furthermore, SENP3 plays a critical role in SUMO2/3 precursor maturation and deconjugation 15. Additionally, SENP5 predominantly cleaves SUMO2, while SENP6 and SENP7 preferentially disassemble SUMO polymers 13 (Table 1).

### Biologically significant manipulation by SUMOylation in tumors

**Figure 2:**
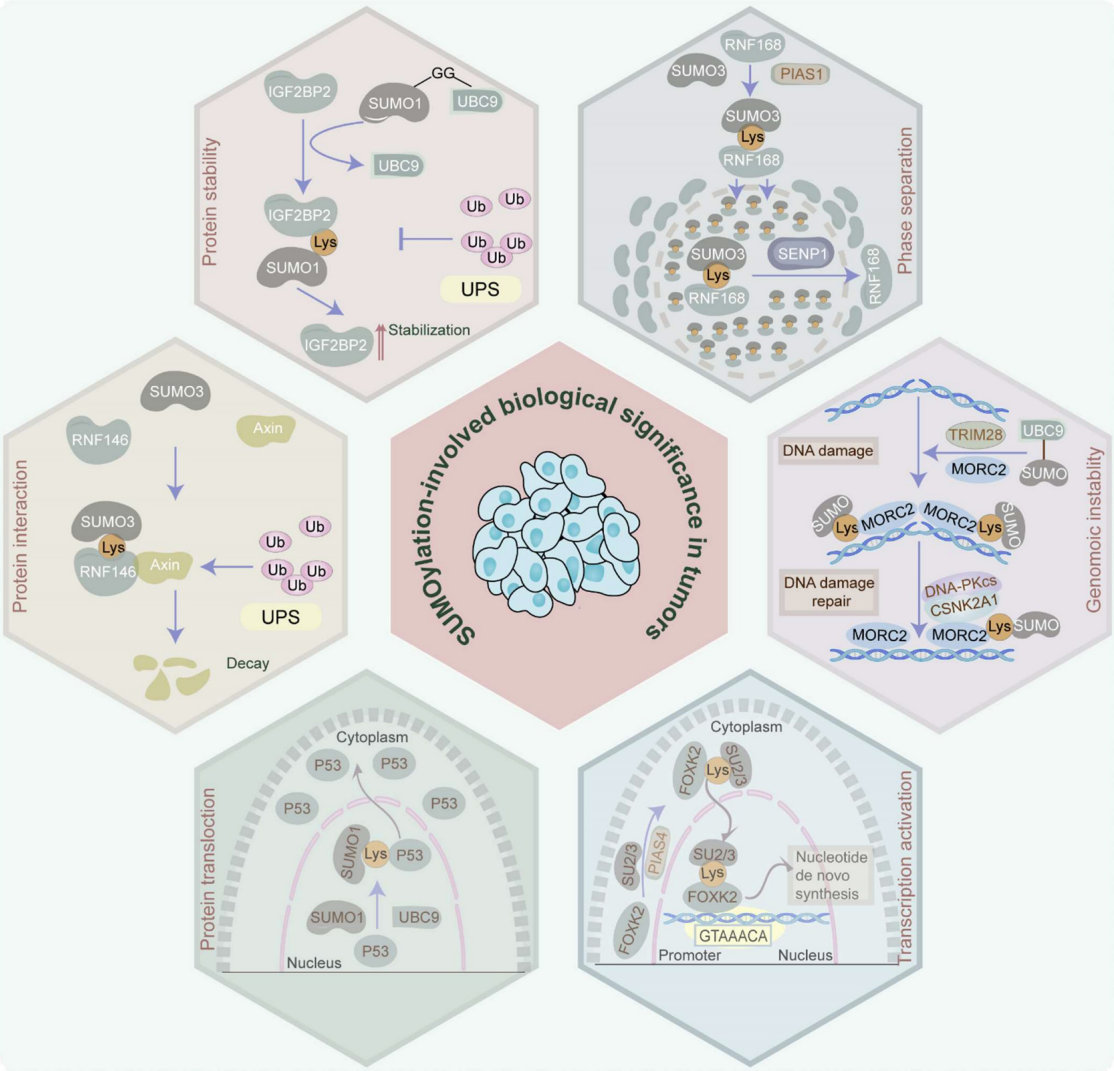
** Biological significance manipulated by SUMOylation in tumors.** Protein SUMOylation governs a spectrum of biological effects in tumors, involving protein stability, protein-protein interaction, protein translocation, phase separation, transcriptional activation, and genomic instability, by which it participates in the disruption of cell homeostasis via inducing aberrance of gene expression, cell cycle progression, signaling pathway transduction, DNA damage response, RNA metabolism, etc.

In tumor cells, protein SUMOylation orchestrates essential molecular processes, predominantly influencing protein stability, protein-protein interaction, protein translocation, phase separation, and transcriptional activity. It also participates in cell homeostasis disruption via aberrant gene expression induction, cell cycle progression, signaling pathway transduction, DNA damage response, and RNA metabolism (Figure 2).

#### Protein stability

SUMO modifiers, which are analogous to ubiquitin, administer protein stability within tumors. Characteristically, SUMOylation blocks the ubiquitin-proteasome system (UPS)-induced protein degradation. Specifically, insulin-like growth factor 2 mRNA-binding protein 2 (IGF2BP2), which is SUMOylated at K497, K505, and K509 by SUMO1, avoids degradation by UPS and enhances its protein stability, thereby facilitating vasculogenic mimicry in glioma 16. Similarly, the SUMOylation of myeloid cell leukemia 1 (MCL1) at K234 and K238 increases its protein stability through the prevention of UPS-mediated MCL1 protein degradation, leading to the proliferation of cancer cells 17. Moreover, SUMO1-mediated SUMOylation of RNA-binding protein Raly (RALY) at K175 improves its protein stability, thereby facilitating vasculogenic mimicry in glioma cells 18. Additionally, the noncovalent attachment of SUMO-2/3 to the SIM (236-240aa) of 5-methylcytosine (m^5^C) RNA methyltransferase NOL1/NOP2/Sun domain family member 2 (NSUN2) enhances its protein stability and induces m^5^C modification, exacerbating gastric cancer 19. Of note, SUMOylation can also facilitate protein degradation via the proteasome pathway in rare cases. For example, SUMO2-mediated SUMOylation of GLUT1 promotes the ubiquitination of GLUT1 in nasopharyngeal carcinoma 20. Additionally, transcription factor CP2c, which is involved in human malignancies, is SUMOylated at its K50 and SIM158 in a SUMO1-dependent manner. The SUMOylated CP2c is subsequently decayed by the ubiquitin-independent PSME3/20S proteasome system 21. These studies confirm the dual role of SUMOylation in maintaining protein stability.

#### Protein-protein interaction

SUMOylation enhances protein-protein interactions which are crucial for tumor initiation and progression. For instance, SUMO3-modified SUMOylation of E3 ubiquitin ligase RING finger protein 146 (RNF146) at K19, K61, K174, and K175 facilitates the association of RNF146 with axis inhibition protein 1 (Axin) to expedite the ubiquitination and degradation of Axin, resulting in the progression of hepatocellular carcinoma (HCC) 22. Similarly, SUMO1-mediated SUMOylation of promyelocytic leukemia (PML) protein at K65, K160, and K490 increases its interaction with proto-oncogene c-Myc (c-Myc) and further stabilizes c-Myc protein, contributing to the deterioration of glioblastoma (GBM) malignancy 23. Furthermore, SUMO-2/3 modification of Flotillin-1 (Flot-1) at K195 boosts its interaction with nuclear zinc finger protein SNAI1 (Snail) and inhibits Snail proteasomal degradation, thereby promoting epithelial-to-mesenchymal transition (EMT) of metastatic prostate cancer 24. Additionally, SUMOylation of OTU domain-containing ubiquitin aldehyde-binding protein 2 (OTUB2) at K233 anchors it to yes-associated protein (YAP)/PDZ-binding motif (TAZ) via a “V/I-X-V/I-V/I” SIM in YAP and TAZ, contributing to tumor metastasis in a Hippo-independent manner 25.

#### Protein translocation

Proteins perform their specific biological functions in different cell sub-regions to help cells accurately respond to different physiological, pathological, or environmental stimuli. Strikingly, SUMOylation regulates the occurrence and development of tumors via altering the location of proteins. Concretely, in HCC, SUMO1-mediated SUMOylation via the IKII_265-268_ SIM site of pyruvate kinase M2 (PKM2) relocates PKM2 from the cytoplasm to the nucleus, leading to glycolytic reprogramming and cancer progression via EMT induction and signal transducer and activator of the transcription 3 (STAT3) signaling pathway 26. Likewise, Ran-binding protein 2 (RanBP2)-mediated SUMOylation of interleukin-33 (IL-33) at K54 facilitates its nuclear shuttling, therefore resulting in immune evasion in HCC 27. Furthermore, in prostate cancer, SUMOylation of tumor suppressor gene p53, which is mediated by the RanBP2/SUMO1/Ubc9 complex, facilitates its translocation from the nucleus to the cytoplasm, promoting malignancy progression 28. Also in prostate cancer, SUMO-2 modification of extracellular-signal-related kinase 5 (ERK5) at K6 and K22 improves the ability of ERK5 to translocate to the nucleus, ultimately facilitating tumor cell proliferation 29. Additionally, SUMOylation influences nuclear translocation by impacting protein phosphorylation. For instance, SUMO1-mediated p65 SUMOylation at K37, K122, K123, and K221 sites increases p65 phosphorylation at the S276 site, and further stimulates the nuclear import of p65 and NF-κB transcriptional activity, ultimately contributing to the malignant phenotype of liver cancer cells 30.

#### Phase separation

Liquid-liquid phase separation (LLPS) is essentially a physical and chemical phenomenon. In living cells, LLPS drives the inhomogeneous spatiotemporal coordination of individual molecules into membrane-free, droplet-like biomolecular condensates (BMCs) 31. Notably, the establishment of LLPS plays a significant role in tumor initiation, progression, metastasis, and drug resistance. In fact, *in vitro* assay demonstrates that the rate of SUMOylation is tremendously improved in the LLPS-driven condensate model that is artificially engineered 32. Furthermore, *in silico* analysis indicates that the strong and weak SUMO-SIM interactions can create conditions for the formation of condensates 33. Additionally, intrinsically disordered region (IDR) is a general hallmark of proteins that participate in LLPS. Meanwhile, the proteomics strategy reveals that lysine residues residing in the disordered regions of proteins are prone to be SUMOylated 34. All these results indirectly prove that SUMOylation may be a driving factor of LLPS. However, whether SUMOylation is the authentic cause of the formation of LLPS needs further proof. Recently, SUMOylation was identified to facilitate the LLPS via specifically targeting lysine residues within the IDRs of proteins in tumors 35. Concretely, SUMOylation of RING finger protein 168 (RNF168) which is triggered by SUMO3 and PIAS family at K210 in a long IDR sequence isolates RNF168 from DNA damage sites, attenuates RNF168-mediated ubiquitination, and sequesters the repair protein TP53-binding protein 1 (53BP1) in nuclear condensates via evoking LLPS, eventually attenuating DNA damage repair efficiency. However, overexpressed SENP1-induced deSUMOylation reverses these biological processes, contributing to drug resistance in colon cancer 6.

#### Transcriptional activation

Transcription factors are indispensable for cancer-related signaling pathways. Of note, SUMOylation represents a dual influence on the transcriptional activation of transcription factors. Typically, SUMO1-mediated SUMOylation of transcription factor forkhead box protein M1 B (FOXM1B) at K463 represses *p21* transcription but facilitates *JNK1* transcription in breast cancer cells, indicating that its key involvement in the transcriptional activity of FOXM1B 36. Moreover, transcription factor (forkhead box K2) FOXK2 which is SUMOylated by SUMO2/3 at K527 and K633 contributes to its nuclear translocation and expedites the transcription of nucleotide synthetic genes, leading to tumorigenesis and chemoresistance in HCC 37. Conversely, SUMO1-mediated SUMOylation of Eyes absent homolog 1 (Eya1) at K43 and K146 hampers its transcription activity in triple-negative breast cancer cells 38.

#### Genomic instability

Genomic instability induced by ectopic DNA damage repair mechanisms is a vital hallmark of tumor occurrence, deterioration, and anti-tumor drug resistance. Remarkably, SUMOylation has also been demonstrated to play a role in DNA damage repair and genome maintenance. For instance, SUMOylation of microrchidia CW-type zinc finger 2 (MORC2) by SUMO1/2/3 and tripartite motif containing 28 (TRIM28) at K767 elevates its interplay with casein kinase II subunit alpha (CSNK2A1), triggering DNA-dependent protein kinase catalytic subunit, therefore stimulating DNA repair and chemoresistance in breast cancer 39. Furthermore, the SUMO2/3-mediated SUMOylation of minichromosome maintenance protein 10 (MCM10) at K669 contributes to the proliferation and metastasis of esophageal squamous cell carcinoma through the induction of aberrant DNA replication licensing and genomic instability 40. Of note, deSUMOylation can also dysregulate the DNA damage repair process. Specifically, SENP5 can deSUMOylate histone H2A.Z (H2AZ) via interacting with K121, K122, and K126 within the DNA binding domains in the C-terminal of H2AZ to facilitate DNA damage repair in a homologous recombination-dependent manner and mediate CRC resistance 41.

In summary, the SUMOylation cascade exerts multifaceted effects in cancer cells, influencing critical processes such as gene expression, cell metabolism, and tumor cell proliferation. Targeting SUMOylation holds promise for developing novel cancer therapeutics, and a comprehensive understanding of SUMOylation provides insights into identifying predictive biomarkers and personalized therapeutic strategies.

## The role of SUMOylation in sustaining malignancy

**Figure 3:**
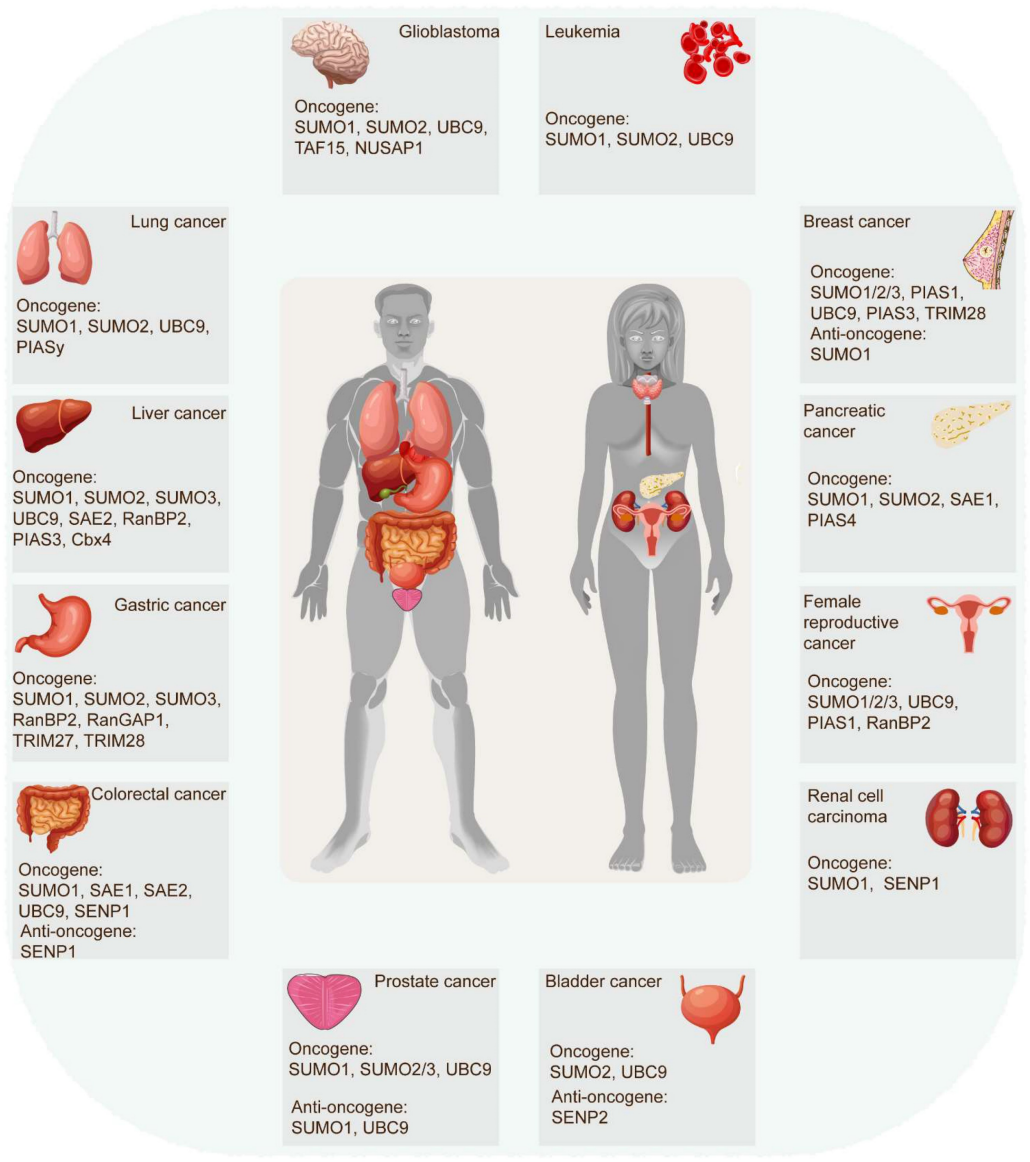
** Role of SUMO molecules in tumors.** SUMOylation cascade is frequently identified in tumor initiation, progression, and response to therapies. In most instances, SUMOylation plays an indispensable role in exacerbating cancer progression. However, some SUMOylation molecules have been proven to act as tumor-suppressive roles in certain human tumors, even playing distinct roles in the same tumor.

**Figure 4:**
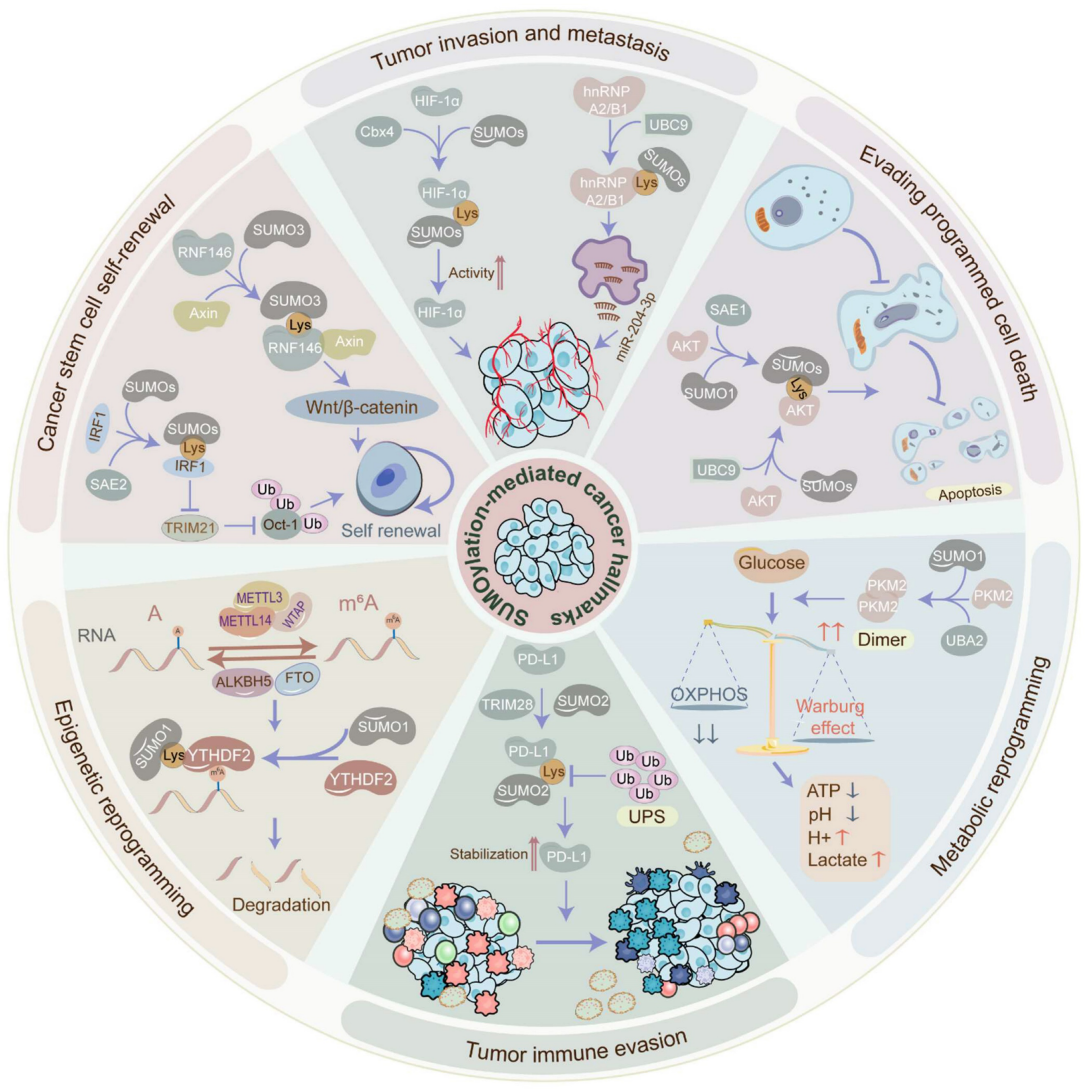
** The biological functions of SUMOylation cascade in tumors.** SUMOylation participates in the acquisition or maintenance of cancer hallmarks, such as tumor invasion, metastasis, cancer stem cell self-renewal, epigenetic reprogramming, immune evasion, metabolic reprogramming, and programmed cell death escape. SUMOylation is involved in tumor invasion and metastasis via administrating tumor angiogenesis, EMT, etc. SUMOylation induces cancer stem cell maintenance and self-renewal via governing cancer stemness-related genes and pathways. SUMOylation manages epigenetic reprogramming via perturbing transcriptional activation, protein interaction, protein localization, protein stability, and RNA metabolism. SUMOylation facilitates tumor immune evasion via altering the tumor microenvironment and abolishing immune surveillance. SUMOylation promotes metabolic reprogramming via stimulating the Warburg effect. SUMOylation enhances the escape of programmed cell death via blocking apoptosis and autophagy. EMT: epithelial-to-mesenchymal transition.

The SUMOylation cascade frequently emerges as a key player in tumor initiation, progression, and response to therapies. In most cases, SUMOylation exerts an indispensable role in driving cancer progression. However, certain SUMOylation-related enzymes and proteins demonstrated tumor-suppressive roles in specific contexts, sometimes even within the same tumor (Figure 3) (Table 2). For example, SUMO1-mediated SUMOylation of p65 recruits mesencephalic astrocyte‐derived neurotrophic factor (MANF) and increases their interaction, thus restraining the NF‐κB/Snail pathway, EMT, and HCC progression 42. Conversely, the SUMO1-mediated SUMOylation of methyltransferase-like 3 (Mettl3) stabilizes *Snail* mRNA in an N6-methyladenosine (m^6^A)-dependent manner, promoting EMT and HCC progression 43. These findings underscore the complexity of SUMOylation's effect on tumorigenicity. Recent evidence increasingly links SUMOylation with tumorigenesis, tumor progression, anti-tumor therapy response, and poor survival. Accumulating research has robustly demonstrated SUMOylation's oncogenic role in tumor invasion and metastasis, cancer stem cell self-renewal, angiogenesis, evasion of programmed cell death, metabolic reprogramming, and more. (Figure 4).

### The role of SUMOylation in tumor invasion and metastasis

The characteristic phenotype of tumor cell dissemination is the invasive-metastatic cascade which is the most lethal factor of tumors. Strikingly, the SUMOylation cascade facilitates tumor metastasis by stimulating tumor angiogenesis, EMT, etc. Tumor angiogenesis is the formation of new blood vessels to provide tumors with sufficient oxygen and nutrients for growth and metastasis.

Thus, tumor angiogenesis inhibition has been speculated as a promising anti-tumor therapeutic strategy. While the role of SUMOylation in tumor angiogenesis is not fully elucidated, some evidence suggests its involvement. For instance, the most representative driven pathway is the vascular endothelial growth factor (VEGF) signal transduction pathway. However, the SUMOylation of VEGF is yet to be verified. Merely one study reports that SUMO1-induced SUMOylation of vascular endothelial growth factor receptor 2 (VEGFR2) at K1270 hampers the activity of VEGFR2 and the angiogenesis signaling pathway 44, indicating an inhibitory effect of the SUMOylation cascade on tumor invasion and metastasis. Conversely, the SUMOylation of hypoxia-inducible factor-1alpha (HIF-1α) can upregulate VEGF in the hypoxic environment to stimulate angiogenesis. For instance, SUMO E3 ligase chromobox protein homolog 4 (Cbx4) boosts the transcriptional activity of HIF-1α via SUMOylating HIF-1α at K391 and K477 under hypoxic conditions, following which HIF-1α further transcriptionally activates VEGF, potentiating angiogenesis in HCC 45. However, SUMOylating of HIF-1α also shows a converse role in VEGF expression. Specifically, hypoxia-mediated SUMOylation of HIF-1α facilitates its binding to E3 ubiquitin ligase and promotes its ubiquitination and degradation in a proline hydroxylation-independent manner, contributing to the downregulation of VEGF 46. Additionally, SUMOylation of heterogeneous nuclear ribonucleoprotein A2/B1 (hnRNP A2/B1) in hypoxic conditions facilitates its translocation from the nucleus to the cytoplasm and further enhances the exosome-sorting process of miR-204-3p via binding to its RRM1 motif segment, expediting angiogenesis in GBM 47.

EMT, characterized by increased invasiveness and metastatic potential, is regulated by SUMOylation in various cancers. For instance, overexpressed nucleolar and spindle-associated protein 1 (NUSAP1), associated with SUMO E3 ligase RanBP2, robustly triggers the SUMOylation of transcription factor 4 (TCF4) and the Wnt/β-catenin signaling pathway, contributing to the advancement of EMT and cervical cancer cell metastasis 48. On the contrary, SUMOylation can hinder the EMT and tumor metastasis. Concretely, SUMO E3 ligase PIAS3-mediated SUMOylation of E3 ubiquitin ligase SMAD specific E3 ubiquitin protein ligase 2 (Smurf2) at K26 and K369 enhances Smurf2 protein stability and further degrades transforming growth factor-β (TGFβ) receptor, thus attenuating EMT, cancer cell invasiveness, and metastasis 49.

Collectively, these findings highlight the dual role of the SUMOylation cascade in tumor angiogenesis and EMT, adding complexity to the regulatory mechanisms governing the invasive-metastatic cascade through SUMOylation.

### The role of SUMOylation in cancer stem cell maintenance and self-renewal

Cancer stem cells (CSCs) represent a small subset of undifferentiated cells within tumor tissues, distinguished by their robust self-renewal and tumorigenic potential. The maintenance of CSCs relies heavily on self-renewal mechanisms, crucial for tumor cell survival and proliferation. Intriguingly, the global SUMOylation level exhibits distinct patterns in CSCs, exerting a notable influence on CSC maintenance and self-renewal. Indeed, the global SUMOylation level in CSCs has been observed to be higher compared to non-CSCs. Mechanistically, SUMOylation of interferon regulatory factor 1 (IRF1), a transcriptional activator of ubiquitin E3 ligase tripartite motif containing 21 (TRIM21), at K78 hinders its transcription activity, leading to reduced expression of TRIM21, thereby attenuating the ubiquitination of Oct-1, which is a transcriptional activator of aldehyde dehydrogenases, and eventually facilitating CSC maintenance and self-renewal in CRC cells 50. Moreover, SUMO1-induced SUMOylation of PML protein augments its interplay with c-Myc, stabilizing c-Myc oncoprotein, and contributes to the CSC self-renewal in glioma 23. Additionally, the SUMOylation cascade has been implicated in expanding the CSC pools in breast cancer and CRC 51.

In addition to manipulating cancer stemness-related proteins, the SUMOylation cascade also engages in the well-known signaling pathways that favor the maintenance and self-renewal of CSCs. Notably, the activation of the Wnt/β-catenin signaling and inhibition of the Hippo signaling are depicted to sustain the self-renewal of stem cells 52, 53. Specifically, E3 ubiquitin ligase RNF146 SUMOylated at K19, K61, K174, and K175 enhances its nuclear localization and the interaction with Axin, facilitating Axin degradation and Wnt/β-catenin signaling activation in HCC 22. Moreover, E3 ubiquitin-protein ligase UHRF2-induced SUMOylation of TCF4 stabilizes TCF4 and activates Wnt signaling in CRC 54. Furthermore, SUMOylation of Large tumor suppressor 1 (Lats1) at K751 undermines the kinase activity, therefore impeding Hippo signaling in tumors 55.

Taken together, the SUMOylation cascade plays a crucial role in maintaining and self-renewal of CSCs, which are essential for tumor initiation, progression, recurrence, and metastasis. This is achieved through the modification of oncoproteins and the regulation of associated pathways. Inhibiting the SUMOylation cascade may hold promise in impairing the self-renewal ability of CSCs.

### The role of SUMOylation in evading programmed cell death

Programmed cell death (PCD) such as apoptosis, autophagy, and ferroptosis is essential for maintaining cellular homeostasis. Remarkably, the SUMOylation cascade contributes to the dysregulation of PCD in cancer. Apoptosis, a highly regulated form of cell death, is committed to removing non-functional, harmful, abnormal, and misplaced cells promptly. SUMOylation cascade has been involved in apoptotic pathways, administrating the destiny of tumor cells. Of note, the AKT signaling pathway, known to inhibit apoptosis in tumors, is directly influenced by SUMOylation. For instance, the SUMOylation of AKT at K276 in the SUMOylation consensus motif dramatically motivates its kinase activity in cancers 56. Similarly, the SUMOylation of AKT at K276 improves AKT kinase activity without influencing its phosphorylation level but directly phosphorylating Ubc9 at Thr35 and SUMO1 at Thr76 to govern the global SUMOylation status, ultimately contributing to tumorigenesis 57. Additionally, SUMO E1 activating enzyme SAE1 facilitates AKT SUMOylation and phosphorylation, aiding in apoptosis evasion in glioma 58. Autophagy is a process of self-degradation to recycle cellular components 59. Dramatically, the SUMOylation cascade also modulates the autophagy of tumor cells. In breast cancer cells, disruption of SUMO1 complexes contributes to autophagy-mediated tumor cell death, potentially through the upregulation of Tribbles pseudokinase-3 (TRIB3) 60. Conversely, the deSUMOylation of m^6^A demethylase AlkB Homolog 5 (ALKBH5) enhances its activity and further increases the stability of *DDIT4* mRNA, inducing autophagy and tumorigenesis in head-neck squamous cell carcinoma (HNSCC) 61. Ferroptosis, an iron-dependent PCD driven by excessive lipid peroxidation, has participated in tumorigenesis and tumor development 62. Increasing evidence indicates that SUMOylation is involved in ferroptosis in cancer. For instance, SENP1-mediated deSUMOylation of A20 disturbs its interplay with Acyl-CoA synthetase long-chain family member 4 (ACSL4) and solute carrier family 7 member 11 (SLC7A11), contributing to the inhibition of ferroptosis in lung cancer 63. Similarly, SENP1-induced deSUMOylation of ACSL4 hinders ferroptosis via decreasing ACSL4 protein stability in HNSCC 64. Additionally, PIAS4-mediated SUMOylation of SLC7A11 at K500 inhibits ferroptosis in breast cancer 65.

Altogether, PCD exerts an essential effect on tumorigenesis, tumor progression, recurrence, and chemoradiotherapy resistance. Therefore, targeting the SUMOylation cascade may offer a promising approach to sensitize tumors to therapy and suppress tumor progression, thereby ameliorating the overall status quo of tumor treatment.

### The role of SUMOylation in metabolic reprogramming

Metabolic reprogramming, the process by which cells adapt their metabolism to support survival and growth, plays a crucial role in malignant transformation and tumor progression. Strikingly, the SUMOylation cascade can determine metabolic reprogramming, such as the Warburg effect and pentose phosphate pathway 66. Warburg effect also known as aerobic glycolysis, a typical abnormality of glucose metabolism in tumors, is manipulated by SUMOylation. Concretely, SUMO1-induced SUMOylation of PKM2 via binding to the SUMO-interacting motif site IKII265-268 promotes PKM2 dimerization and nuclear translocation, leading to glycolysis in HCC 26. Similarly, IKII265-268-mediated SUMOylation of PKM2 conduces to its distribution into ectosomes, inducing the metabolic reprogramming in monocytes and reshaping the tumor microenvironment 67. Furthermore, a novel circRNA circRNF13 which is downregulated in nasopharyngeal carcinoma diminishes the expression of SUMO2 and the SUMOylation level of glucose transporter type 1 (GLUT1), finally inhibiting GLUT1 degradation and promoting glycolysis 20. However, the SUMOylation cascade can restrain glycolysis in tumors. Hexokinase 2 (HK2), the first rate-limiting enzyme of glycolysis, is SUMOylated at K315 and K492 in prostate cancer cells, restraining its binding to the mitochondria, thereby reducing the tumor cell glycolysis 68. In addition to aerobic glycolysis, SUMOylation may also dysregulate the pentose phosphate pathway in tumors. Specifically, glucose-6-phosphate dehydrogenase (G6PD), a rate-limiting enzyme in the pentose phosphate pathway, is SUMOylated in a SUMO1-dependent manner, therefore enhancing G6PD protein stability and promoting the progression of clear-cell renal cell cancer (ccRCC) 69.

Overall, these findings highlight the crucial role of the SUMOylation cascade in regulating metabolic reprogramming in tumors, exerting both promoting and inhibitory effects. Hence, targeting the SUMOylation cascade may therefore offer a promising strategy to suppress metabolic reprogramming, improve the tumor microenvironment, inhibit tumor growth, and enhance the sensitivity of anti-tumor drugs.

### The role of SUMOylation in tumor immune evasion

Tumor immune evasion, characterized by the ability of tumor cells to evade immune surveillance, is a major factor in tumor progression and immunotherapy resistance. Functionally, the SUMOylation cascade expedites tumor immune evasion via altering the tumor microenvironment and abolishing immune surveillance. Aerobic glycolysis, a central factor in regulating the tumor microenvironment, is conducive to tumor immune evasion as well 70. Of note, the SUMOylation cascade regulates antigen presentation deficiency and lymphocyte inactivation. For example, SUMO2/3-mediated SUMOylation of scaffold attachment factor B (SAFB) at K294 declines the abundance and activity of tumor-infiltrating T cells and blocks MHC class I antigen presentation, inducing immune evasion in cancer 71. Moreover, UBC9-mediated SUMOylation of signal transducer and activator of transcription 4 (STAT4) at K350 attenuates its nuclear translocation and stability, further blocking the pro-inflammatory activation of macrophages and impeding the anti-tumor T cell response in prostate cancer 72. Furthermore, cholesterol-induced SUMOylation of liver X receptors (LXRs) hinders IL-9 expression via inhibiting p65-IL-9 binding, contributing to the reduction of IL-9-producing CD8+ T cell differentiation and anti-tumor response 73. In addition to immune cells, SUMOylation occurring in tumor cells can also facilitate tumor immune evasion. Specifically, SUMOylation of programmed cell death protein-1 ligand (PD-L1) by TRIM28, an E3 ubiquitin ligase and E3 SUMO ligase, stabilizes PD-L1 via hampering PD-L1 ubiquitination and enhancing PD-L1 SUMOylation, leading to the T cell inactivation and immune evasion in gastric cancer 74. Additionally, E3 ligase RanBP2-induced SUMOylation of nuclear factor IL-33 at K54 prevents the degradation of transcription factor IRF1 which elevates the expression of PD-L1, a T cell immune checkpoint ligand, in HCC cells, ultimately leading to the inactivation of T cells and immune surveillance 27.

In conclusion, immunotherapy represents a novel paradigm in cancer therapy. However, a significant number of patients with tumors remain unresponsive to this new anti-tumor strategy. Notably, the SUMOylation cascade contributes to tumor immune evasion, indicating that targeting SUMO modification could enhance anti-tumor immunity. Combining tumor immunotherapy with SUMOylation inhibitors may offer a promising approach to overcoming resistance to immunotherapy.

### The role of SUMOylation in epigenetic reprogramming

Epigenetic reprogramming, encompassing transcriptional factor regulation, RNA epigenetic modification, protein translational modification, etc., orchestrates changes in cell fate through epigenetic modifications without altering the DNA sequence 75. Of interest, SUMOylation plays a pivotal role in manipulating epigenetic reprogramming, impacting transcriptional activation, protein interaction, protein localization, protein stability, and RNA metabolism as mentioned above. Moreover, SUMOylation, as a form of epigenetic modification itself, participates in the dysregulation of epigenetic reprogramming in cancers, fostering tumor heterogeneity. At the transcriptional level, SUMOylation governs gene expression via SUMOylating transcription factors. For example, SUMO1-induced SUMOylation of the transcription factor nuclear factor erythroid-2 related factor 2 (NRF2) at K110 stimulates the transcription of glutathione peroxidase 2 (Gpx2) and motivates the intracellular reactive oxygen species (ROS)-phosphoglycerate dehydrogenase (PHGDH) signaling, promoting the *de novo* serine synthesis and tumorigenesis in HCC 76. Notably, histone SUMOylation is also significant for gene expression. For instance, SUMO1-induced SUMOylation of histone H4 upregulates the expression of the progesterone receptor (PR) gene, promoting the proliferation of Ishikawa Cells and inhibiting the apoptosis of tumor cells in endometrial cancer 77. Moreover, SUMOylation can manipulate gene expression post-transcriptionally influencing the m^6^A and m^5^C modifications. Specifically, SUMO1-mediated SUMOylation of the m^6^A reader YT521-B homology domain-containing family protein 2 (YTHDF2) at K571 improves its binding affinity for m^6^A-modified mRNAs, facilitating cancer progression 78. Strikingly, the SUMOylation of the E3 ubiquitin ligases can boost their interaction with substrate proteins. For instance, SUMO3-mediated SUMOylation of RING-type E3 ubiquitin ligase RNF146 at K19, K61, K174, and K175 enhances the association between RNF146 and its substrate Axin, expediting the ubiquitination and degradation of Axin and consequently deteriorating the progression of HCC 22. Likewise, deubiquitinating enzymes can be SUMOylated to promote tumor progression. For instance, SUMOylation of the deubiquitinating enzyme OTUB2 at K233 heightens its interaction with YAP/TAZ and stabilizes YAP/TAZ via deubiquitination, contributing to cancer metastasis 25.

Collectively, elucidating the molecular mechanisms of SUMOylation-mediated epigenetic reprogramming is crucial for uncovering new interventions to conquer tumor heterogeneity.

## Cellular stresses that induce SUMOylation in cancers

**Figure 5:**
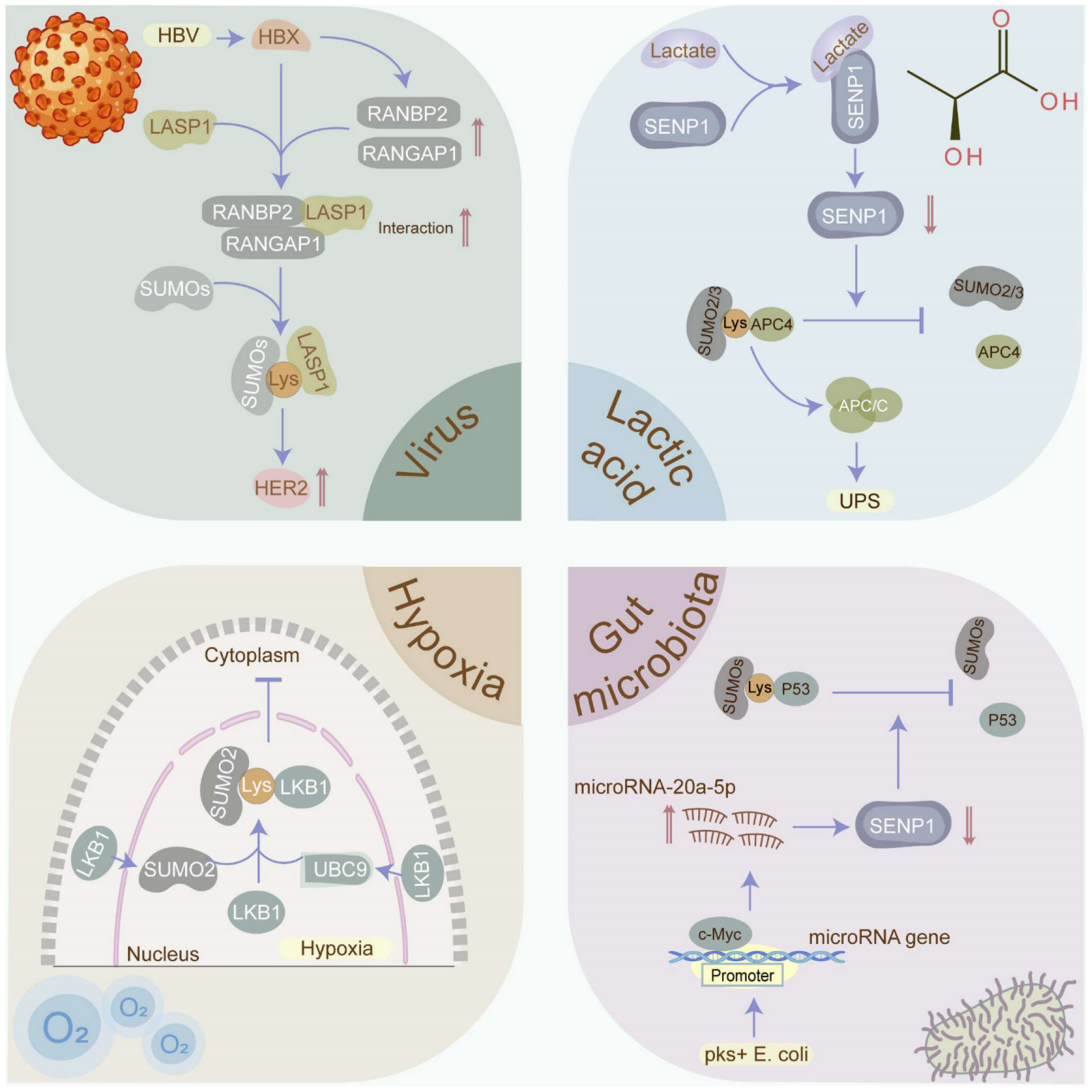
** Cellular stressors that induce SUMOylation in cancers.** SUMOylation which plays a critical role in maintaining cellular homeostasis is evoked by diverse cellular stresses, including viruses, hypoxia, gut microbiota, and lactic acid. Viruses such as HBV, EBV, and HPV have been confirmed to contribute to cancers via manipulating the SUMOylation process. Hypoxia has been identified as the inducement the SUMOylation in tumors. Gut microbiota and its metabolites such as pks+ E. coli and SCFAs participate in the tumorigenesis via modulating the SUMOylation in p53 and NF-κB signaling. Lactate may promote tumor progression via stabilizing the anaphase-promoting complex (APC/C) in a SUMOylation-dependent manner. SCFA: short-chain fatty acid.

Protein SUMOylation, critical for maintaining cellular homeostasis, is evoked by diverse cellular stresses. Nevertheless, what cellular stresses can provoke SUMOylation and how SUMOylation responds to them remain enigmas. Herein, we delve into the stimuli and mechanisms underlying SUMOylation induction in cancers (Figure 5).

### Hypoxia and SUMOylation

Hypoxia, a prevalent feature in solid tumors, is closely related to tumor proliferation, differentiation, angiogenesis, energy metabolism, anti-tumor drug resistance, and poor prognosis. Remarkably, hypoxia has been identified as a trigger for SUMOylation 79. Yet, how hypoxia sustains the malignant phenotypes via activating SUMOylation requires further elaboration. Of note, hypoxia-inducible factors (HIFs), indispensable regulators of the cellular response to hypoxia, link hypoxia to SUMOylation-mediated tumorigenesis. Specifically, the SUMOylation of E3 ligase HAF induced by hypoxia specifically facilitates HIF-2 binding to the DNA promoters and driving transcription, thus leading to the growth and metastasis of ccRCC 80. In addition to HIFs, hypoxia-mediated SUMOylation is responsible for cancer progression via supervising other transcription activators or transcriptional repressors. In lung cancer, hypoxia facilitates SUMOylation-dependent competition between transcriptional repressor HIC1 and transcription activator Sp1, leading to the transcriptional repression of SIRT1 and subsequently promoting tumor metastasis 81. Analogously, hypoxia promotes the SUMOylation of transcriptional repressor Slug via diminishing its interaction with SUMO proteases, contributing to lung cancer metastasis 4. Moreover, hypoxia-induced SUMOylation impairs the biogenesis pathway of tumor suppressor microRNAs, promoting tumorigenesis and cancer progression. For instance, SUMO1-mediated SUMOylation of KH-type splicing regulatory protein (KHSRP) modified at the site K87 in the hypoxia microenvironment segregates KHSRP from the pri-miRNA/Drosha-DGCR8 complex and disturbs the transformation of pre-miRNAs from pri-miRNAs, contributing to the inhibition of TL-G-Rich microRNA biogenesis and the advancement of tumorigenesis 82. Furthermore, hypoxia-induced SUMOylation could manipulate the RNA epigenetic modification such as m^6^A.

Concretely, hypoxia facilitates the cancer progression via inducing the SUMOylation of YTHDF2 78. Additionally, SUMOylation in response to hypoxic stress can regulate tumorigenesis and progression via altering protein localization. In liver cancer, liver kinase B1 (LKB1) which is SUMOylated by SUMO-2 at Lys178 under a hypoxic microenvironment expedites tumor growth via impeding LKB1 nucleocytoplasmic shuttling 83. Intriguingly, *in vivo* research has been utilized to intervene in intratumoral hypoxia, with hyperoxic breathing at 60 % O_2_ demonstrated to reverse hypoxia in tumor microenvironments and hamper the expression of HIF-1α and its downstream target proteins 84. However, it is yet to be determined whether hyperoxic breathing manipulates the SUMOylation process.

In summary, SUMOylation is a hypoxia-related post-translational modification. Targeting intratumoral hypoxia, such as by improving tumor oxygenation, may reduce the overall SUMOylation levels, thereby hindering cancer progression.

### Viruses and SUMOylation

Approximately 10% of cancers are associated with virus infections, principally including Epstein-Barr virus (EBV), human papilloma virus (HPV), hepatitis B virus (HBV), etc. 85. Intriguingly, these viruses contribute to carcinogenesis in part via manipulating the SUMOylation process. EBV, a known human oncogenic virus, consistently infects individuals globally, opportunistically leading to tumorigenesis. Strikingly, EBV promotes tumorigenesis partially through EBV-mediated SUMOylation. In EBV-positive lymphomas, the EBV oncoprotein latent membrane protein-1 (LMP1) triggers NF-κB signaling to elevate the levels of SUMOs 86, simultaneously hijacking SUMO-conjugating enzyme UBC9 87 and diminishing SUMO-protease SENP2 activity and turnover 88, collectively contributing to the enhancement of overall SUMOylated protein levels and tumorigenesis. Similarly, HPV facilitates the malignant transformation of the host's infected cells via administrating SUMOylation. For instance, HPV E6/E7 oncoproteins up-regulate SUMO-conjugating enzyme UBC9 via inhibiting the autophagy-lysosome pathway to govern SUMOylation during HPV-mediated tumorigenesis and induce apoptosis evasion 89. Likewise, HBV infection plays a significant role in hepatocarcinogenesis via controlling SUMOylation. Mechanistically, HBV X (HBX) protein facilitates the SUMOylation of LASP1 through the RANBP2-RANGAP1 complex and enhances the interaction between LASP1 and human epidermal growth factor receptor 2 (HER2) to upregulate HER2 by hampering ubiquitination-mediated proteasomal degradation, resulting in hepatocarcinogenesis 90.

Given the distinctive capability of viruses to manipulate the SUMO pathway and enhance tumorigenesis, the eradication of oncoviruses through vaccination may impede their capacity to increase global cellular SUMOylation and induce malignant transformation.

### Gut microbiota and SUMOylation

Emerging evidence suggests a connection between the gut microbiota and its metabolites with multiple malignancies. Escherichia coli harboring the pks island (pks+ E. coli) has been closely implicated in the tumorigenesis and progression of CRC. Mechanistically, pks+ E. coli transcriptionally activates miR20a-5p which hijacks SUMO-protease SENP1 and further declines SENP1 expression, ultimately reducing the p53 SUMOylation and colon tumor growth 91. Multiple research indicates that NF-κB signaling is a canonical mechanism for inflammation-associated colorectal tumorigenesis. A recent study has validated that the SUMOylation in intestinal cells, which prevents IκBα degradation and hinders the NF-κB signaling, is triggered by the short-chain fatty acids (SCFAs) produced by gut microbiota in a pH-dependent manner, thus attenuating the inflammatory responses and maintaining the intestinal epithelial integrity 92. However, the abundance of SCFAs and SCFA-producing bacteria is tremendously shrunk in CRC 93, with low fecal SCFA concentration associated with a higher CRC incidence 94.

These findings suggest that gut microbiota and its metabolites may promote colorectal tumorigenesis and progression through the modulation of SUMOylation, leading to the activation of the NF-κB signaling pathway or disruption of the p53 pathway in intestinal cells. Interventions targeting the gut microbiota and its metabolites, such as SCFA-producing probiotics and dietary fiber supplementation, hold promise for inhibiting tumor initiation and progression.

### Tumor metabolite and SUMOylation

Lactic acid, considered as the most representative tumor metabolite and traditionally viewed as a metabolic waste product of aerobic glycolysis or Warburg effect in cancers, has emerged as a significant oncometabolite. Lactic acid is exported to the tumor microenvironment (TME) to shape an environment with accumulated lactate and decreased pH value, promoting invasion, metastasis, angiogenesis, immune evasion, etc. 95. Strikingly, a recent study highlighted that cumulative lactate can stabilize the anaphase-promoting complex (APC/C) via mediating SUMOylation to expedite cell proliferation. Mechanistically, increasing lactate directly interacts with zinc in the SUMO protease SENP1 active site to induce SENP1 inhibition and manipulate the activity of E3 ubiquitin ligase APC/C, resulting in vigorous cell proliferation 96. Meanwhile, APC/C has been validated to play the role of oncoprotein in cancers principally via degrading substrate proteins such as Axin 97 and SMAR1 98. Comprehensively, we speculate that lactate produced via the Warburg effect may facilitate the SUMOylation of APC/C, contributing to tumorigenesis and progression by targeting multiple tumor suppressors. Moreover, lactate-driven SUMOylation may extend beyond APC/C, necessitating further exploration of potential substrate proteins. Thus, decreasing lactate levels or inversing the Warburg effect may reduce the global SUMOylation level in cancers, providing a novel anti-tumor strategy.

## Therapeutic potential and toxicity of SUMOylation inhibitors

The proteins involved in the SUMOylation enzymatic cascade not only sustain cancer hallmarks but also diminish the sensitivity of anti-tumor therapies. Of note, dysregulation of SUMOylation-related proteins such as SAE1/2, UBC9, SUMO E3-ligases, and SENPs has been observed in multiple cancers 24, 74, 99, leading to anti-tumor drug resistance via fortifying tumor stemness 50, promoting DNA damage repair 6, and provoking nucleotide de novo synthesis 37, etc. Consequently, targeting these enzymes in the SUMOylation cascade presents an attractive anti-tumor strategy 100, 101. Over the past two decades, a plethora of natural and synthetic SUMOylation inhibitors have been identified (Table 3). However, except for TAK-981, all the SUMOylation inhibitors are in the preclinical research and development stage.

### SUMO E1 inhibitors

SUMO E1, which functions as activating enzymes in the form of SAE1/2 dimer, has been linked to cancer exacerbation. For instance, upregulation of SAE1 or SAE2 facilitates tumor proliferation, metastasis, or apoptosis evasion in intrahepatic cholangiocarcinoma (ICC) 99, HCC 102, glioma 58, and lung cancer 103, etc. So far, several SUMO E1 inhibitors have been discovered or synthesized. The foremost discovered SAE1/2 inhibitors include ginkgolic acid extracted from Ginkgo biloba leaves, anacardic acid, and kerriamycin B from microbial metabolites 104, 105. These natural compounds impede protein SUMOylation via blocking the formation of the SAE1/2-SUMO intermediate and the conjugation of SUMOs to substrates. Remarkably, *in vivo* and *in vitro* studies have corroborated that exposure to ginkgolic acid or anacardic acid inhibits the proliferation, migration, and apoptosis escape of GC, breast cancer, CRC, etc. 51, 106. Subsequently, two novel natural compounds were identified, namely Davidiin (extracted from the plant Davidia involucrate) 107 and tannic acid (purified from Gallotannin) 108, as SUMO E1 inhibitors, sharing analogous mechanisms of action with previously discovered inhibitors. Experimentally, both inhibitors restrain the growth of multiple cancer cells, involving GC MKN-45 cells, prostate cancer DU-145 cells, and lung cancer NCI-H460 cells 107, highlighting their anti-tumor ability. However, these natural compounds lack efficiency and specificity due to the half maximal inhibitory concentration (IC50) ranging in micromole and wide spectrum of targets.

There is therefore an immediate demand for inhibitors targeting the SUMO E1 enzymes that are both highly efficient and highly specific. To date, synthetic E1 inhibitors, which predominantly include compound-21 109, COH000 110, ML-792 111, ML-93 112, and TAK-981 113, have garnered attention. Mechanistically, compound-21 occupies the ATP binding site of SUMO E1 and further hampers the formation of the E1-biotinylated SUMO-1 thioester intermediate. COH000 covalently binds to the Cys30 of Uba2 and concomitantly induces structural changes of SUMO E1, shaping an inactive conformation. In 2017, ML-792 was authenticated to selectively decline SAE1/2 enzyme activity and global SUMOylation level via establishing an adduct with SUMO in an ATP-dependent manner catalyzed by the enzyme itself 111. In acute myeloid leukemia, ML-792 treatment induces the deconjugation of all the SUMO-2/3 targets and blunts Daunorubicin-mediated transcriptional reprogramming 114. ML-93 as the derivative of ML-792 manifests a robust selectivity to hinder the SUMOylation via an identical mechanism of action in pancreatic cancer, contributing to G2/M phase arrest and apoptosis 112.

Notably, a great breakthrough in targeting the SUMO pathway is TAK-981, a derivative of ML-792. Mechanistically, TAK-981 impedes the activity of SUMO-activating enzyme via shaping a SUMO-TAK-981 adduct in the enzyme catalytic site 113.

Of note, *In vivo* and* ex vivo* assays have demonstrated that TAK-981 directly evokes anti-tumor immune responses via the pharmacological reactivation of IFN1 signaling 115-118. Specifically, TAK-981 facilitates T cell sensitivity, macrophage phagocytosis, and NK cell cytotoxicity. A recent study revealed that TAK981 disrupts immune suppressive activities of regulatory T (Treg) cells in an IFN-alpha receptor 1 (IFNAR1)-dependent manner 119. Moreover, TAK981 can restrain trogocytosis and increase the viability of endogenous and immunotherapeutic cytotoxic T cells via upregulating cholesterol 25-hydroxylase (CH25H) 120. Furthermore, TAK-981 effectively motivates the antitumor immune responses via driving T cell activation and augmenting the percentage of activated CD8 T cells and natural killer (NK) cells in preclinical models 115, 118. Additionally, an *in vivo* study reported that TAK-981 in combination with the anti-CD20 antibody rituximab can potentiate macrophage phagocytosis and NK cell cytotoxicity in lymphoma models 116. These studies demonstrate that the SUMOylation inhibitor TAK981 plays a crucial role in the recuperation of immune-killing competence. In addition to type 1 interferon and immune-dependent mechanism, TAK-981 also provokes apoptosis and cell-cycle arrest in acute myeloid leukemia 121. Additionally, TAK-981 dampens the expression of SUMOylated hnRNP A2/B1 and total hnRNP A2/B1, further thwarting exosome sorting of miR-204-3p as well as resulting in the prohibition of tumor growth and angiogenesis in GBM 47.

Strikingly, multiple phase 0/1/2 clinical trials of TAK-981 have been implemented. For instance, a phase 0 clinical trial (NCT04065555) was utilized to evaluate the biological effects on the TME of intratumoral TAK-981 injection and TAK-981 injection combined with cetuximab or avelumab in 12 patients with head and neck carcinoma, revealing that both can switch the TME from immune-suppressive to immune-permissive in an IFN pathway-dependent manner 122. Moreover, a phase 1/2 clinical trial (NCT03648372) was performed to assess the safety, tolerability, efficacy, and pharmacokinetics of TAK-981 in patients with hematologic malignancies or solid tumors. Furthermore, another three TAK-981 combination medication studies (namely NCT04381650, NCT04074330, and NCT04776018) are already undergoing phase 1 clinical trials. NCT04381650 (TAK-981 in combination with pembrolizumab) focuses on the safety, tolerability, and anti-tumor activity of combination medications in patients with solid tumors, while NCT04074330 (TAK-981 in combination with rituximab) evaluates the safety of the drug and the efficacy of combination medications in patients with CD20-positive non-Hodgkin lymphoma. Additionally, NCT04776018 (TAK-981 in combination with anti-CD38 monoclonal antibodies) is centered on the safety and efficacy of combination medications in patients with multiple myeloma. However, these clinical trials either have limitations due to the small number of recruited patients or failure to produce any substantial results.

Collectively, these *in vitro*, *in vivo*, and clinical studies reveal that natural and synthetic compounds targeting SUMO E1, especially TAK-981, perturb the state of tumor cells and the TME and impede tumorigenesis and cancer progression, thereby offering a new approach for anti-tumor treatment.

### SUMO E2 inhibitors

UBC9, as the sole SUMO E2 conjugating enzyme, receives SUMO proteins delivered by E1 and cooperates with E3 to identify the substrates. In most cases, UBC9 acts as an oncogene. Therefore, silencing UBC9 has the potential to restrain proliferation and migration and facilitate apoptosis of tumor cells, such as osteosarcoma U-2OS cells 123. Of note, several SUMO E2 inhibitors have been screened out, involving Spectomycin B1, 2-D08, GSK145A, etc. Spectomycin B1, a natural compound derived from Streptomyces spectabilis initially recognized as an antibiotic against gram-positive bacteria, straightly attaches to UBC9, specifically impeding the establishment of the E2-SUMO intermediate and leading to the proliferative inhibition of MCF7 human breast-cancer cells 124. Similarly, 2-D08, a synthetic oxygenated flavonoid, blocks the conjugation of SUMOs from the UBC9-SUMO thioester to substrates, thereby hindering SUMOylation in tumor cells 125, such as MDA-MB-231 breast-cancer cells 60. Although GSK145A is not a specific noncovalent inhibitor of UBC9-dependent SUMOylation, it acts as a competitive substrate of SUMOylation, blocking the substrate recognition for Tricho-rhino-phalangeal syndrome type I protein (TRPS1) 126. Compound 2, a synthesized UBC9-binding compound identified by small-molecule microarray (SMM)-based screening using fluorescently tagged UBC9, prevents the SUMOylation of RanGAP1 127. Additionally, WNN0605-F008 disturbs the catalytic activity of UBC9 via occupying its catalytic pocket, leading to the inhibition of RanGAP1 SUMOylation 128.

Altogether, the advancement of high-throughput screening assays has led to the discovery of inhibitors for the “undruggable” E2 enzyme UBC9. However, their clinical value requires further evaluation.

### SUMO E3 inhibitors

SUMO E3-ligases ensure the specificity of target substrates and facilitate the transfer of SUMOs from the E2 conjugating enzyme to the substrates. Unfortunately, small-molecule inhibitors for SUMO E3-ligases are scarce. UNC3866 is one such inhibitor that binds preferentially to the CBX chromodomains of SUMO E3-ligase CBX4, inhibiting PC3 prostate cancer cells 129. Additionally, UNC3866 exerts a powerful anti-tumor effect via hindering CBX4, contributing to the suppression of tumor cell growth and cancer stem cell properties 130. Further development of SUMO E3 inhibitors with enhanced specificity is needed to disrupt the SUMOylation cascade effectively.

### SUMO protease inhibitors

SUMO proteases act as both oncogenes and anti-oncogenes. In some tumors, the high expression of partial SUMO proteases exacerbates the malignant phenotypes, making them anti-tumor targets. Therefore, several natural and synthetic small-molecule inhibitors targeting SENP1 and SENP2 have been identified. For instance, streptonigrin, a natural product, directly binds to SENP1, thwarting its interaction with SUMO1 and leading to the diminution of hypoxia-inducible factor alpha (HIF1α) 131. Triptolide, another natural small-molecule inhibitor, augments the cellular SUMOylation in prostate cancer cells via mitigating the mRNA and protein levels of SENP1, contributing to the inhibition of tumor cell growth *in vivo* and *in vitro* 132. Similarly, a natural product Momordin Ic reduces prostate cancer cell proliferation and induces cell apoptosis via decreasing SENP1 expression and disrupting its SUMO2-RanGAP1-induced cleavage 133. Correspondingly, several synthetic small-molecule compounds, such as GN6958 134, compound 3 135, and compound 13m 136, have also been developed to inhibit SENP1 activity; however, further verification is warranted using *in vivo* studies. Furthermore, three SENP2 inhibitors have been developed, namely ebselen 137, Compound 69 and 117 138. However, there is no evidence that these SENP2 inhibitors exert anti-tumor properties.

Taken together, SUMO proteases play dual roles in the SUMOylation cascade, involving promoting the SUMO maturation and expediting the SUMO deconjugation. Thus, inhibiting SUMO proteases may induce the antithetical outcomes of the SUMOylation cascade. Therefore, the anti-tumor effect of SUMO protease inhibitors requires not only *in vivo* and *in vitro* verification but also further clinical validation.

An* in vivo* study reported that loss of Ubc9 in adult mice can lead to severe diarrhea or death 139, indicating that SUMOylation inhibitors may possess potential toxicity and induce adverse effects. Moreover, the unanticipated tumor-suppressive role of SUMOylation may have significant implications for the appropriate use of SUMOylation inhibitors in clinical practice 140.

In summary, while most SUMOylation inhibitors hold promise in anti-tumor treatment, their potential toxicity and unexpected side effects necessitate further investigation. Considering the dual role of SUMOylation in tumors and physiological homeostasis, it is crucial to assess the safety and efficacy of these inhibitors comprehensively before their clinical use.

## Conclusions and perspective

SUMOylation, as one of the critical PTMs, modulates protein function and localization, exerting tremendous influence on tumor initiation and progression. SUMOs are covalently conjugated to the lysine (K) residues of proteins with a SUMOylation consensus motif. To date, more than 3,600 SUMOylated proteins, with at least 7,300 SUMOylation sites, have been authenticated utilizing mass spectrometry and bioinformatics technology 141, underscoring the pivotal impact of this dynamic and reversible SUMOylation cascade on protein fate. To date, the SUMOylation process has been depicted adequately after about 30 years of in-depth research. However, there are still some enigmas in this multi-step enzymatic cascade. First, why do such a small series of SUMO enzymes catalyze thousands of protein substrates? Second, what exactly is SUMO5? a human SUMO pseudogene or a functional isoform? Third, what other cellular stress can induce SUMOylation in tumors? Fourth, what specific functions does each SUMO E3 ligase perform? Will there be new SUMO E3 ligases identified? Fifth, why does SUMOylation degrade substrate protein in some cases? Is this protein degradation induced by specific SUMO E3 ligase-mediated SUMOylation? With the development of multi-omics sequencing and deep learning, a better understanding of protein SUMOylation will be provided in future years.

In tumors, the SUMOylation cascade possesses the characteristic of exacerbating the cancer progression relying on its multifunctional biological effects, such as regulating transcriptional activity, increasing protein stability, etc. Intriguingly, SUMOylation can be triggered by various cellular stresses, involving hypoxia, viral infections, gut microbiota, and lactic acid accumulation, indicating that interfering with these cellular stresses holds promise for inhibiting tumor progression. However, the specific mechanisms of SUMOylation induced by cellular stresses remain unclear. Additionally, SUMOylation, as a form of non-mutational epigenetic reprogramming, engages in the management of various cancer hallmarks. Increasing evidence has successively illustrated that SUMOylation assists tumor cells in proliferation, invasion, metastasis, angiogenesis, PCD escape, metabolic reprogramming, and immune evasion. Thus, targeting enzymes involved in the SUMOylation cascade emerges as a promising anti-tumor strategy, with multiple small-molecule inhibitors targeting SUMOylation enzymes identified. However, merely one small-molecule inhibitor has progressed to clinical trials, highlighting the need for further research to address potential adverse effects using advanced techniques such as virtual high-throughput screening, molecular docking, proteolysis targeting chimera (PROTAC), and molecular glue. By leveraging multidisciplinary collaboration, the concurrent application of SUMOylation inhibitors in anti-tumour interventions holds the promise to impede tumor invasion, metastasis, and recurrence, and overcome drug resistance and immune evasion, ultimately advancing tumor therapeutics.

## Figures and Tables

**Table 1:** Molecules and molecular characteristics in the SUMO pathway

Subsets	Enzymatic activity	Molecules	Molecular weights	Cellular location
**SUMO**	None	SUMO1	11.56 kDa	Nucleus, cytoplasm
		SUMO2	10.87 kDa	Nucleus
		SUMO3	11.64 kDa	Cytoplasm, nucleus
		SUMO4	10.65 kDa	Nucleus
		SUMO5	11.53 kDa	Nucleus
**E1**	Activating SUMO	SAE1	38.45 kDa	Nucleus
		SAE2	71.22 kDa	Cytoplasm, nucleus
**E2**	Conjugating SUMO	UBC9	18.00 kDa	Nucleus, cytoplasm
**E3**	Ligating SUMO	*SP-RING Family*		
		PIAS1	71.84 kDa	Nucleus
		PIAS2	68.24 kDa	Nucleus
		PIAS3	68.02 kDa	Cytoplasm, nucleus
		PIAS4	56.50 kDa	Nucleus
		MMS21	27.93 kDa	Nucleus
		*SIM Family*		
		RANBP2	358.20 kDa	Nucleus
		KIAA1586	89.67 kDa	Cytoplasm, nucleus
		CBX4	61.37 kDa	Nucleus
		ZNF451	121.48 kDa	Nucleus
		SLX4	200.01 kDa	Nucleus
		*TRIM Family*		
		TRIM1	83.21 kDa	Cytoplasm
		TRIM27	58.49 kDa	Nucleus, cytoplasm, mitochondrion
		TRIM28	88.55 kDa	Nucleus
		TRIM38	53.42 kDa	Cytoplasm
		PML	97.55 kDa	Nucleus, cytoplasm
**SUMO protease**	SUMO precursor	SENP1	73.48 kDa	Nucleus, cytoplasm
	maturation,	SENP2	67.86 kDa	Nucleus, cytoplasm
	deconjugating	SENP3	65.01 kDa	Nucleus, cytoplasm
	SUMO	SENP5	86.69 kDa	Nucleus
		SENP6	126.15 kDa	Nucleus
		SENP7	119.67 kDa	Cytoplasm

**Table 2:** The function and mechanism of SUMO Molecules in cancers

Cancer type	SUMO enzyme	Substrate	SUMOylation sites	Function and mechanism	Refs
Bladder cancer	SUMO2, UBC9	hnRNPA2B1	K108	promote lymphatic metastasis via driving TBK1 mRNA circularization	142
	SUMO2	DDX39B	K53	promote lymph node metastasis via driving nuclear export of circNCOR1	143
	SUMO2, UBC9	hnRNPA1	K113	promote lymph node metastasis via packaging ELNAT1 into EVs	144
	SENP2	TGF-βRI	K389	inhibit EMT and metastasis via deSUMOylatiing TGF-βRI	145
BRCA	SUMO1/2/3, TRIM28	MORC2	K767	promote chromatin remodelling and DNA repair	39
	UBC9	SYNJ2BP-COX16	K107	promote tumor progression via DRP1-mediated mitochondrial fission	146
	SENP1	HIF-1α	K391, K477	promote metastasis via activating HIF-1 signalling	147
	UBC9, PIAS3	Rac1	K183, K184, K186, K188	promote metastasis via activating Rac1	148
	PIAS1	DDX5	K53	promote tumor progression via forming DDX5/Drosha/DGCR8 complex	149
	SUMO1/2/3, PIAS1	TFAP2A	K10	promote tumor outgrowth via inhibiting transcriptional activity of TFAP2A	150
	SUMO1	Eya1	K43, K146	inhibit tumorigenesis via repressing Eya1 transcription activity	38
	SUMO1	FOXM1	K201, K218, K460, K478, K495	delay mitotic transition via enhancing APC/Cdh1-mediated ubiquitination	151
Cervical cancer	SUMO1, RanBP2	TCF4	Unknown	promote metastasis via activating Wnt/β-catenin signaling	48
	SUMO1/2/3, UBC9, PIAS1	FoxM1b	Unknown	inhibit tumorigenesis by inducing destabilization and cytoplasmic localization of FoxM1b	152
CRC	SAE1, SAE2, UBC9	IRF1	K78	promote maintenance and self-renewal of cancer stem cells via stabilizing Oct-1	50
	SUMO1	IQGAP1	K1445	enhance tumorigenesis and tumor progression via activating AKT-ERK signaling	153
	SENP1	RNF168	K210	promote DNA damage repair via decreasing RNF168 phase separation	6
	SENP1	ELOC	K32	promote stemness via facilitating the deubiquitination and stabilization of HIF1A	154
	SENP1	p16, p19, p21, p27	Unknown	facilitate tumor growth via downregulating CDK inhibitors	155
ESCC	SUMO2/3	MCM10	K669	promote metastasis via inducing genomic instability	40
	SUMO2/3	HSP27	Unknown	promote tumor progression via upregulating PKM2	156
GC	TRIM28	TBK1	K63	promote immune escape via increasing PD-L1 abundance	74
	SUMO2/3	NSUN2	SIM (236-240aa)	promote tumor progression via regulating mRNA m5C methylation	19
	SUMO2/3, RanBP2, RanGAP1	DAXX	Unknown	promote tumor progression via modulating the subcellular localization of DAXX	157
	SUMO1	p38α	K152	promote metastasis via activating MK2 and accelerating ROS accumulation	158
	SUMO1	hnRNP K	K422	promote tumorigenicity and metastasis via stabilizing β-catenin	159
	SUMO2/3	hnRNPM	Unknown	promote tumor progression via regulating alternative splicing	160
	SUMO1, TRIM27	TUFT1	K79	promote tumor progression via activating AKT/mTOR signaling	161
GBM	SUMO1	PML	K65, K160, K490	facilitate malignancy via stabilizing c-Myc protein	23
	NUSAP1	ATR	Unknown	promote tumor progression via suppressing the ubiquitin-dependent proteolysis of ATR	162
	SUMO1, UBC9	CYLD	K40	promote proneural-to-mesenchymal transition via unleashing NF-κB signaling	163
	SUMO2, TAF15	NOP58	Unknown	promote stem cell maintenance and tumorigenicity via regulating 2'-O-methylation	164
	UBC9	hnRNP A2/B1	K108	promote angiogenesis via eliminating miR-204-3p	47
	SUMO1	CDK6	K216	promote tumor progression via stabilizing CDK6 protein	165
HCC	Cbx4	HIF-1α	K391, K477	promote angiogenesis via enhancing VEGF expression	45
	SUMO1, UBC9	PEPCK1	K124	promote tumor progression via mediating glucose metabolism	166
	SUMO1	Lats1	K751	promote tumor progression via inhibiting Hippo signaling	55
	SUMO2	LKB1	K178	promote tumor progression via altering LKB1 localization	83
	SUMO1	NRF2	K110	maintaining tumorigenesis via promoting de novo serine synthesis	76
	SUMO1, UBC9	Mettl3	K177, K211, K212, K215	promote tumor progression via increasing Snail expression in an m6A-dependent manner	43
	RANBP2	FTO	K216	promote tumorigenesis via regulating m6A modification	5
	SUMO1, SAE2	PKM2	SIM (IKII265-268)	promote tumor progression and metastasis via inducing the Warburg effect	26
	RanBP2	IL-33	K54	promote immune escape via stabilizing IRF1	27
	SUMO3, UBC9, PIAS3	RNF146	K19, K175	promote tumor progression via degrading Axin and activating Wnt/β-catenin signaling	22
	SENP1	EIF3I	K298	promote metastasis via inducing EMT	167
HNSCC	SENP1	ACSL4	Unknown	promote tumor progression via inhibiting ferroptosis	64
	SENP1	STAT1	K110, K703	promote tumor progression	168
ICC	SUMO1, SAE1	AKT	Unknown	promote tumor progression	99
	UBC9	p27kip1	Unknown	promote tumorigenesis via governing p27kip1 nuclear export	169
Leukemia	SUMO1	PKM2	K270	inhibit myeloid differentiation via degrading RUNX1	170
	SUMO1, UBC9	IGF-1R	K1025, K1100	promote proliferation of leukemia cells	171
	SUMO1	sPRDM16	K568	promote tumor progression	172
	SUMO2	ERG	K37, K74, K289	promote tumor progression via enhancing ERG stability	173
Lung cancer	SUMO1, UBC9	YTHDF2	K571	promote tumor progression by increasing binding affinity of YTHDF2 with m6A-modified mRNAs	78
	SUMO2, UBC9	hnRNPA2B1	K108	promote lymphatic metastasis via modulating extracellular vesicles	174
	SUMO1, UBC9, PIASy	Slug	K239, K240, K244, K248, K258	promote metastasis via enhancing the transcriptional repression activity of Slug	4
	SUMO1	KEAP1	K39	promote tumor growth via increasing NRF2-target gene expression	175
PDAC	SUMO2, SAE1	hnRNPA1	K113	promote lymphangiogenesis via packaging hnRNPA1 into the extracellular vesicle	176
	SUMO1, PIAS4	VHL	Unknown	promote tumor growth via activating hypoxia signaling	177
Prostate cancer	UBC9	STAT4	K350	inhibit immunostimulatory macrophage activation and antitumor T cell response via hindering transcriptional activity of STAT4	72
	SUMO1, UBC9	HK2	K315, K492	inhibit tumorigenesis via decreasing glycolysis	68
	SENP1	HIF-1α	Unknown	promote progression and metastasis via HIF1α signaling	178
	SUMO2/3, UBC9	Flot-1	K51, K195	promote EMT via inhibiting Snail degradation	24
RCC	SUMO1	HAF	K94, K141	promote growth and metastasis via activating HIF-2	80
	SENP1	HIF2α	Unknown	promote metastasis via inducing EMT	179

**Table 3:** Inhibitors of SUMO molecules

Compound	Target	Cancer type	Compound source	Compound type	Refs
Ginkgolic acid	E1	Gastric cancer, Breast cancer, Uveal melanoma	Ginkgo biloba leaves	Alkylphenol	104
Anacardic acid	E1	Thyroid cancer, Nonpromyelocytic acute myeloid leukemia, Breast cancer, Colon cancer, B-cell lymphoma	Microbial metabolites	Analog of ginkgolic acid	104
Kerriamycin B	E1	Unknown	Microbial metabolites	Antibiotic	105
Davidiin	E1	Gastric cancer, Prostate cancer, Lung cancer	Davidia involucrate	Ellagitannin	107
Tannic acid	E1	Unknown	Gallotannin	Gallotannin	108
Compound-21	E1	Unknown	Synthetic	Phenyl urea	109
COH000	E1	Unknown	Synthetic	Dimethyl 1-((R)-1-(phenylamino)-2-(p-tolyl)ethyl)-7-oxabicyclo [2.2.1]hepta-2,5-diene-2,3-dicarboxylate	110
ML-792	E1	Hepatocellular carcinoma, Pancreatic cancer	Synthetic	Pyrazole- carbonylpyrimidine	111
ML-93	E1	Pancreatic cancer	Synthetic	Derivative of ML-792	112
TAK-981	E1	Acute myeloid	Synthetic	Derivative of ML-792, Pyrazole- carbonylpyrimidine	113
		Leukemia, Hepatocellular carcinoma, Chronic lymphocytic leukemia, Glioblastoma, Pancreatic cancer, Multiple myeloma			
Spectomycin B1	E2	Nasopharyngeal carcinoma	Streptomyces spectabilis	Antibiotic	124
2-D08	E2	Prostate cancer, Acute myeloid leukemia	Synthetic	Oxygenated flavonoid	125
GSK145A	E2	Unknown	Synthetic	Unknown	126
Compound 2	E2	Unknown	Synthetic	Pyridine	127
WNN0605-F008	E2	Unknown	Synthetic	Heterocycle	128
UNC3866	E3, CBX4	Hepatocellular carcinoma	Synthetic	Unknown	129
Streptonigrin	SENP1	Unknown	Streptomyces flocculus	Antibiotic	131
Triptolide	SENP1	Prostate cancer	root of Tripterygium wilfordii	Tripterygium wilfordii Hook F	132
Momordin Ic	SENP1	Acute myeloid leukemia, Colon cancer, Prostate cancer	Kochia scoparia	Triterpenoid glycoside	133
GN6958	SENP1	Unknown	Synthetic	Phenyl urea	134
Compound 3	SENP1	Unknown	Synthetic	Phenyl	135
Compound 13m	SENP1	Unknown	Synthetic	Phenyl	136
Ebselen	SENP2	Unknown	Synthetic	Organo-selenium	137
Compound 69	SENP2	Unknown	Synthetic	Oxadiazoles	138
Compound 117	SENP2	Unknown	Synthetic	Oxadiazoles	138

## Abbreviations

SUMO: small ubiquitin-like modifier
CRC: colorectal cancer
SENP: sentrin-specific protease
UBC9: ubiquitin carrier protein 9
SIM: SUMO-interacting motif
UPS: ubiquitin-proteasome system
MCL1: myeloid cell leukemia 1
IGF2BP2: insulin-like growth factor 2 mRNA-binding protein 2
RALY: RNA-binding protein Raly
m^5^C: 5-methylcytosine
NSUN2: NOL1/NOP2/Sun domain family member 2
RNF146: RING finger protein 146
Axin: axis inhibition protein 1
HCC: hepatocellular carcinoma
PML: promyelocytic leukemia
c-Myc: proto-oncogene c-Myc
Flot-1: flotillin-1
Snail: zinc finger protein SNAI1
EMT: epithelial-to-mesenchymal transition
OTUB2: OTU domain-containing ubiquitin aldehyde-binding protein 2
YAP: yes-associated protein
TAZ: PDZ-binding motif
PKM2: pyruvate kinase M2
STAT3: signal transducer and activator of the transcription 3
RanBP2: Ran-binding protein 2
IL-33: interleukin-33
ERK5: extracellular-signal-related kinase 5
LLPS: liquid-liquid phase separation
BMC: biomolecular condensate
RNF168: RING finger protein 168
PIAS: protein inhibitors of activated signal transducer and activator of transcription
53BP1: TP53-binding protein 1
FOXM1B: forkhead box protein M1 B
JNK1: Jun amino-terminal kinases 1
FOXK2: forkhead box K2
Eya1: eyes absent homolog 1
MORC2: microrchidia CW-type zinc finger 2
TRIM28: tripartite motif containing 28
CSNK2A1: casein kinase II subunit alpha
MCM10: minichromosome maintenance protein 10
H2AZ: histone H2A.Z
MANF: mesencephalic astrocyte‐derived neurotrophic factor
Mettl3: methyltransferase-like 3
m^6^A: N6-methyladenosine
VEGF: vascular endothelial growth factor
VEGFR2: vascular endothelial growth factor receptor 2
HIF-1α: hypoxia-inducible factor-1alpha
Cbx4: chromobox protein homolog 4
hnRNP: heterogeneous nuclear ribonucleoprotein
NUSAP1: nucleolar and spindle-associated protein 1
TCF4: transcription factor 4
Smurf2: SMAD specific E3 ubiquitin protein ligase 2
TGFβ: transforming growth factor-β
CSC: cancer stem cell
IRF1: interferon regulatory factor 1
TRIM21: tripartite motif containing 21
Lats1: large tumor suppressor 1
PCD: programmed cell death
HNSCC: head-neck squamous cell carcinoma
TRIB3: tribbles pseudokinase-3
ALKBH5: AlkB Homolog 5
GLUT1: glucose transporter type 1
HK2: hexokinase 2
SAFB: scaffold attachment factor B
LXRs: liver X receptors
PD-L1: programmed cell death protein-1 ligand
IFNAR1: IFN-alpha receptor 1
NRF2: nuclear factor erythroid-2 related factor 2
Gpx2: glutathione peroxidase 2
ROS: reactive oxygen species
PHGDH: phosphoglycerate dehydrogenase
YTHDF2: YT521-B homology domain-containing family protein 2
KHSRP: KH-type splicing regulatory protein
LKB1: liver kinase B1
EBV: Epstein-Barr virus
HPV: human papilloma virus
HBV: hepatitis B virus
LMP1: latent membrane protein-1
HER2: human epidermal growth factor receptor 2
SCFA: short-chain fatty acid
TME: tumor microenvironment
APC/C: anaphase-promoting complex
ICC: intrahepatic cholangiocarcinoma
TRPS1: tricho-rhino-phalangeal syndrome type I protein
BRCA: breast cancer
GC: gastric cancer
ESCC: esophageal squamous-cell carcinoma
PDAC: pancreatic ductal adenocarcinoma
RCC: renal cell cancer
